# Intermittent hypoxia causes histological kidney damage and increases growth factor expression in a mouse model of obstructive sleep apnea

**DOI:** 10.1371/journal.pone.0192084

**Published:** 2018-02-01

**Authors:** Bisher Abuyassin, Mohammad Badran, Najib T. Ayas, Ismail Laher

**Affiliations:** 1 Department of Medicine, Faculty of Medicine, University of British Columbia, Vancouver, BC, Canada; 2 Departments of Pharmacology and Therapeutics, Faculty of Medicine, University of British Columbia, Vancouver, BC, Canada; University Medical Center Utrecht, NETHERLANDS

## Abstract

Epidemiological studies demonstrate an association between obstructive sleep apnea (OSA) and accelerated loss of kidney function. It is unclear whether the decline in function is due to OSA per se or to other confounding factors such as obesity. In addition, the structural kidney abnormalities associated with OSA are unclear. The objective of this study was to determine whether intermittent hypoxia (IH), a key pathological feature of OSA, induces renal histopathological damage using a mouse model. Ten 8-week old wild-type male CB57BL/6 mice were randomly assigned to receive either IH or intermittent air (IA) for 60 days. After euthanasia, one kidney per animal was paraformaldehyde-fixed and then sectioned for histopathological and immunohistochemical analysis. Measurements of glomerular hypertrophy and mesangial matrix expansion were made in periodic acid–Schiff stained kidney sections, while glomerular transforming growth factor-β1 (TGF-β1), connective tissue growth factor (CTGF) and vascular endothelial growth factor-A (VEGF-A) proteins were semi-quantified by immunohistochemistry. The antigen-antibody reaction was detected by 3,3′-diaminobenzidine chromogen where the color intensity semi-quantified glomerular protein expression. To enhance the accuracy of protein semi-quantification, the percentage of only highly-positive staining was used for analysis. Levels of TGF-β, CTGF and VEGF-A proteins in the kidney cortex were further quantified by western blotting. Cellular apoptosis was also investigated by measuring cortical antiapoptotic B-cell lymphoma 2 (Bcl-2) and apoptotic Bcl-2-associated X (Bax) proteins by western blotting. Further investigation of cellular apoptosis was carried out by fluorometric terminal deoxynucleotidyl transferase (TdT) dUTP Nick-End Labeling (TUNEL) staining. Finally, the levels of serum creatinine and 24-hour urinary albumin were measured as a general index of renal function. Our results indicate that mice exposed to IH have an increased glomerular area (by 1.13 fold, p< 0.001) and expansion of mesangial matrix (by 1.8 fold, p< 0.01). Moreover, the glomerular expressions of TGF-β1, CTGF and VEGF-A proteins were 2.7, 2.2 and 3.8-fold higher in mice exposed to IH (p< 0.05 for all). Furthermore, western blotting protein analysis demonstrates that IH-exposed mice express higher levels of TGF-β1, CTGF and VEGF-A proteins by 1.9, 4.0 and 1.6-fold (p< 0.05 for all) respectively. Renal cellular apoptosis was greater in the IH group as shown by an increased cortical Bax/Bcl-2 protein ratio (p< 0.01) and higher fluorometric TUNEL staining (p< 0.001). Finally, 24-hr urinary albumin levels were higher in mice exposed to IH (43.4 μg vs 9.7 μg, p< 0.01), while there were no differences in serum creatinine levels between the two groups. We conclude that IH causes kidney injury that is accompanied by glomerular hypertrophy, mesangial matrix expansion, increased expression of glomerular growth factors and an increased cellular apoptosis.

## Introduction

Intermittent hypoxia (IH) is a key pathological feature of obstructive sleep apnea (OSA), the most common sleep related breathing disorder [1]. Epidemiological studies show that patients with OSA suffer an accelerated decline in kidney function. However, it is unclear whether this is due to OSA *per se* or to confounding factors such as obesity, hypertension, diabetes or other concomitant disorders [2–7]. A rodent model of OSA was developed in 2001 that simulates moderate to severe OSA in clinical settings [8], and has been used to investigate the systemic effects of OSA such as insulin resistance [9], endothelial vascular dysfunction [10], and alterations in tumor-associated macrophages function [11]. Importantly, the pathological pattern of IH is distinct from the pathological feature of sustained hypoxia, where IH promotes excessive production of reactive oxygen species and inflammatory mediators, and increases sympathetic activity [12].

With respect to kidney disease, OSA enhances the activity of the renin angiotensin-aldosterone system [13], increases the activity of the sympathetic nervous system [14] and generates systematic and local reactive oxygen species [15]; these alterations are known to induce functional and structural kidney damage. For instance, focal segmental glomerulosclerosis (FSGS) is an important histopathological component of impaired renal function [16] that can result from several metabolic and haemodynamic factors including a diabetic milieu, increased blood pressure and mitochondrial oxidative stress [17–19]. Mechanistically, mesangial matrix expansion (MME) is the cornerstone of FSGS [16–17]. Moreover, excessive accumulation of glomerular extracellular basement membrane (mesangium) is driven by an over- production of a number of growth factors including transforming growth factor-β1 (TGF-β1), connective tissue growth factor (CTGF) and vascular endothelial growth factor-A (VEGF-A) [20–22]. However, the histopathological alterations of the kidney in response to IH have not been reported. We tested the hypothesis that IH alters glomerular structure and modulates the expression of glomerular growth factors.

## Materials and methods

### Animals

After approval from the University of British Columbia Animal Care Committee, ten 8-week old wild-type male CB57BL/6 mice were obtained from JAX Laboratories and allowed to acclimatize for one week with free access to water and regular chow before initiating the IH procedure.

### IH protocol

Animals were randomly assigned to receive either IH or intermittent air (IA) for 60 days as we described previously [10]. Briefly, mice assigned to receive IH were placed in specially designed cages containing oxygen sensors for measuring the fraction of oxygen inspired (FIO_2_) in the cages. The cages were connected to a gas regulator that allowed the flow of sufficient amounts of nitrogen gas to reduce FIO_2_ to 8% within 30 seconds, after which the gas regulator allowed for a rapid replacement of the nitrogen by oxygen and causing the FIO_2_ in the cage to be restored to 21% (room air) within 30 seconds. This 1-minute IH cycle was repeated 60 times per hour for a total of 12 day-light hours per day.

### Animal monitoring and euthanasia

Animals were monitored twice a week for the following parameters: body weight, activity, appearance, breathing and posture/gait. A score of 1 or 2 (see S1 Table) in any category resulted in increased monitoring, including daily weighing, supportive care (e.g. supplemental heat, SQ fluid replacement, gel food or food treats/moistened pellets). A score of 3 in any category or a cumulative score of >5 after appropriate supportive care resulted in immediate euthanasia. Animals were euthanized by inhaling an anesthetic followed by carbon dioxide.

### Kidney sectioning

After 60 days of either IH or IA, mice were euthanized and the right kidney from each animal was fixed in 4% paraformaldehyde (Sigma-Aldrich, Germany) for 24 hours and then sectioned (5 μm) for histopathological and immunohistochemical studies.

### Histopathological assessment

Two paraffin-embedded kidney samples from each group were sectioned (4 kidney sections per slide) and stained with hematoxylin and eosin, periodic acid–Schiff (PAS) and Masson's trichrome stains for pathological evaluation. Sections were evaluated by a certified pathologist who was blinded to the study protocol (i.e. blinded to identity of IA or IH samples) for evaluation of glomerular congestion, matrix expansion and pelvic inflammation.

### Glomerular and mesangial area calculation

Following deparaffinization, kidney sections were stained with PAS for assessment of the total glomerular tuft area and glomerular basement membrane (mesangium) area. The mean glomerular tuft area was determined by counting all available glomeruli (where the glomerular vascular pole is evident) within each kidney section, where one kidney section was used from each animal. The mesangial matrix area was calculated as the PAS-positive area compared to the total glomerular tuft area using 50 randomly selected glomeruli per kidney section (see below). Images were taken at 400x magnification using an Aperio ScanScope**®** CS microscope slide scanner, and measurements of glomerular tuft area were made using Aperio ImageScope (V 12.1.0.5029) software.

### Glomerular selection for area measurements and protein expression

**Fig 1:**
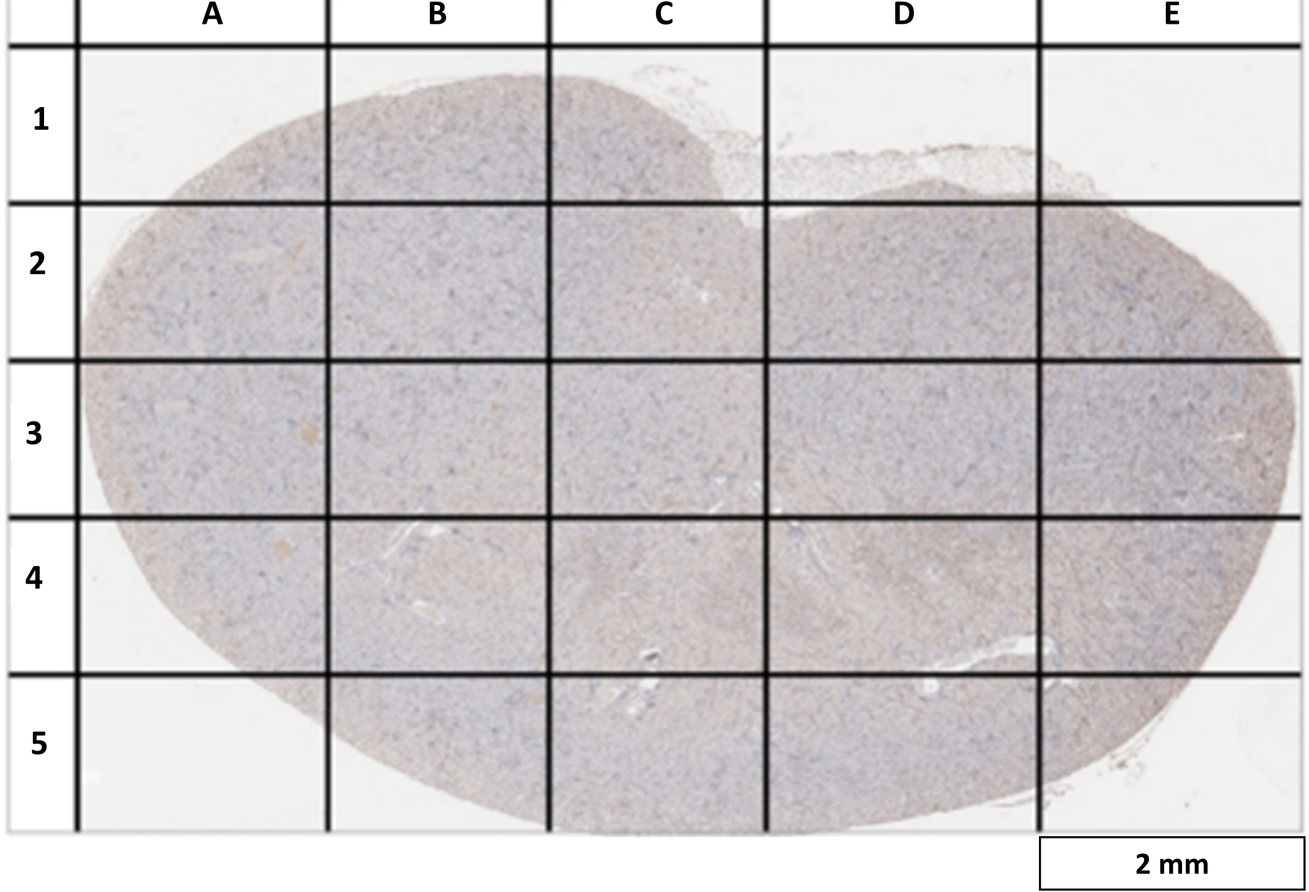
Virtual grid for random glomerular selection in kidney sections. A virtual grid was drawn over a kidney section taken at low magnification (40x). This was used to maximize the glomerular selection area and to minimize selection bias. A minimum of two glomeruli were randomly chosen from each sector so that the total number of glomeruli selected for protein analysis is 50 per kidney sample.

We created a virtual grid with 25 different slots to enable sampling of all areas of each kidney section (Fig 1). A minimum of two different glomeruli were randomly selected from each slot for mesangial area measurements and protein semi-quantification. As some slots did not include any glomeruli (example: slot E1 in Fig 1), the total number of missing glomeruli from such slots was calculated, allowing for a similar number of glomeruli to be reinvestigated so that we were able to sample 50 glomeruli per kidney section.

### Immunohistochemistry

Sections of kidney tissues were deparaffinised and rehydrated using xylene and downgraded concentrations of alcohol, after which slides were immersed in 10 mM sodium citrate (pH 6) for 20 minutes in a steam bath (95 °C) to allow for antigen retrieval. Slides were then incubated in 3% hydrogen peroxidase (Sigma-Aldrich–Germany) for quenching of endogenous peroxidase activity, and non-specific proteins binding was blocked using 1.5% normal blocking serum for 90 minutes. Kidney sections slides were then incubated in goat anti-mouse TGF-β1 (1:50 sc-146-G), CTGF (1:50 sc-14939) and VEGF-A (1:50 sc-152-G) primary antibodies (Santa Cruz Biotechnology–USA) for 24 hours at 4°C. After several washes, slides were incubated with avidin biotinylated horseradish peroxidase-labeled secondary antibody as per manufacturer instructions (ImmunoCruz™ goat ABC Staining System: sc-2023). Finally, slides were stained with 3,3′-diaminobenzidine (DAB) chromogen for protein detection and quantification, and counterstained with hematoxylin for detection of nuclei. Images were taken at 400x magnification using an Olympus BX61 electron microscope. Only 50 images of randomly selected glomeruli were considered per kidney section, and images were analyzed using IHC Profiler plugin [23] within ImageJ free software, which measures the intensity of the brown (DAB) color caused by the antigen-antibody reaction. The percentage of only “highly-positive” brown staining in each glomerulus was measured, so reducing the possibility of other colors influencing the brown stain and thus enhancing the accuracy of protein quantification. The % of highly-positive staining was averaged from 50 different glomeruli per kidney section (to give n = 1).

### Western blotting

Upon euthanasia, 20 mg of the left kidney cortex of each animal was harvested and homogenized in RIPA lysis buffer (sc-24948), sonicated thoroughly and then kept frozen (-80 degrees) for protein analysis. A total of 50 μg total protein from each tissue homogenate was used for protein electrophoresis separation on 12% precast polyacrylamide gel (Bio-Rad: 4568044) as per manufacturer instructions. Proteins were then transferred to nitrocellulose membranes (Bio-Rad: 1620115) and incubated with rabbit anti-mouse 1:1000 β-actin (cell signaling: 4967), 1:1000 GAPDH (Cell signaling: 2118), 1:100 TGF-β1 (sc-130348), 1:100 CTGF (sc-365970), 1:100 VEGF (sc-7269), 1:100 hypoxia inducible factor-1α (HIF-1α) (sc-13515), 1:100 Bax (sc-7480) and 1:100 Bcl-2 (sc-7382) primary antibodies overnight at 4 degrees. After several washes, the membrane was incubated for 1 hour in horseradish peroxidase-conjugated anti-rabbit secondary IgG (cell signaling: 7074S) for β-actin and GAPDH proteins detection, and with horseradish peroxidase-conjugated mouse IgG kappa binding protein (sc-516102) for detection of other mouse monoclonal antibodies. Finally, 1 ml of Clarity™ Western ECL Blotting Substrates (Bio-Rad: 1705060) was added to the membrane for chemiluminescent signaling detection, and images were taken by ChemiDoc™ XRS+ System device (Bio-Rad: 1708265). Images were analyzed by Imagej software and reported as the pixels’ intensity of a target protein relative to either β-actin or GAPDH housekeeping proteins.

### Fluorometric *In situ* cell death detection

Paraffin-embedded kidney sections were deparaffinized and rehydrated as described earlier. Slides were then incubated in phosphate-buffered saline with tween-20 (PBST) solution for 10 minutes for the purpose of permeabilization. After washing with PBS, 50 μl of TUNEL reaction mixture (Roche Applied Science– 12156792910, USA) was added to each kidney section, and sections were then incubated at 37 °C for 1 hour in a humidified chamber. After several washes, slides were counterstained with 4',6-diamidino-2-phenylindole (DAPI) stain for detection of nuclei and finally mounted. Fluorescence images were taken with Olympus BX61 electron microscope at 200x. Four random images were taken from each kidney section to give n = 1, with one kidney section per animal. The number of TUNEL staining-positive cells was normalized to the number of available cells in each image, and averaged from 4 images per kidney section. Cell counting was performed using cell counter plugin within ImageJ free software.

### Renal function index

At the end of study protocol, each mouse was kept separately in a metabolic cage for 24-hour urine collection. Urine samples were kept at -20 degrees for total urinary albumin excretion analysis. Total urinary albumin was quantified using a mouse albumin ELISA kit (41-ALBMS-E01) as per manufacturer instructions. Serum creatinine was measured by a creatinine enzymatic assay (Crystal Chem: 80350) as per manufacturer instructions.

### Statistical analysis

Data are presented as mean ± SD. The normal distribution for each set of the results was tested using the Shapiro-Wilk test of normality; an unpaired t-test was used to calculate differences between groups using GraphPad Prism (Version 6) statistical software, and p values less than 0.05 were considered significant.

## Results

### Animal characteristics

Some animals showed signs of minor distress during the initial phase of exposure to IH, but no signs of illness were observed in any animals during the study period. There were no differences in kidney weight, body weight or fasting blood sugar for IH and IA groups. Details of animal characteristics at the end of the study are shown in Table 1.

**Table 1:** Animal characteristics.

Animal	Kidney weight (gm)	Body weight (gm)	Kidney to body ratio	Fasting blood sugar* (mmol/L)
**IA1**	0.157	26.1	0.0060	3.4
**IA2**	.158	27.3	0.0057	5.6
**IA3**	.142	27.5	0.0052	5.3
**IA4**	.135	27.3	0.0049	5.8
**IA5**	.139	26.0	0.0053	6.3
**IH1**	0.143	28.2	0.0051	5.4
**IH2**	0.139	28.8	0.0048	4.4
**IH3**	0.129	27.2	0.0047	5.2
**IH4**	0.133	24.8	0.0054	3.7
**IH5**	0.147	26.3	0.0056	5.7

IA: Intermittent air, IH: Intermittent hypoxia

*: based on 8-hour overnight fasting

### Histopathological assessment

**Fig 2:**
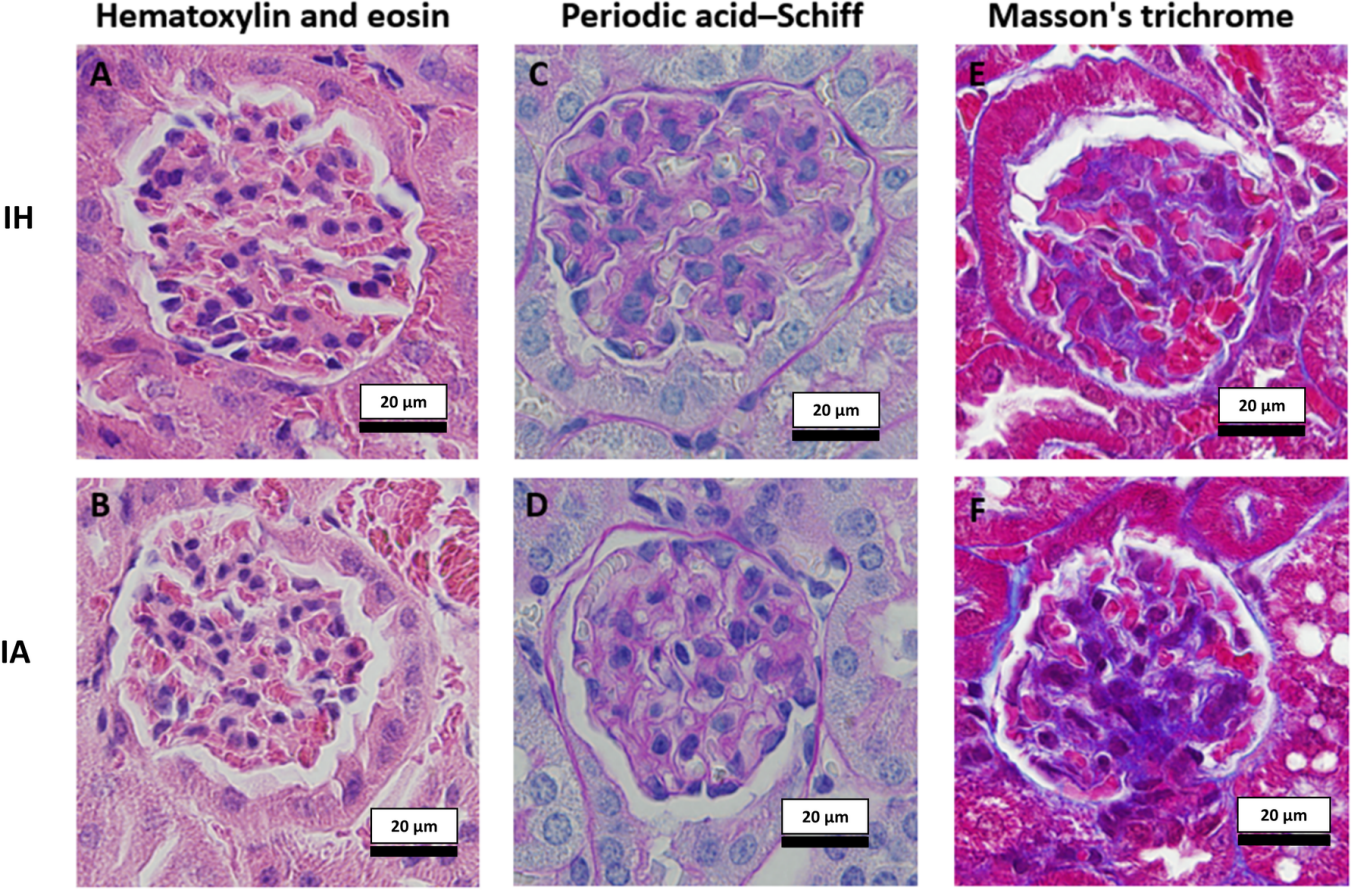
Examples of stained kidney sections used for subjective histological evaluation. Two randomly chosen slides (4 sections per slide) from each group were evaluated by an independent histopathologist after being stained with hematoxylin and eosin (2A (IH), 2B (IA)) for general glomerular and tubular morphological assessment, cellularity of the glomerulus, and for cellular infiltrates in the cortex and medulla. Glomeruli stained with periodic acid–Schiff stain for glomerular basement membrane and mesangium assessment, and for assessment of glomerular capillary loops and tubular epithelium are shown in Fig 2C (IH) and 2D (IA). Assessments of glomerular and tubular collagen and fibrous tissue were made using Masson's trichrome stain; 2E (IH), 2F (IA). Images taken at 400x with standardized light exposure. IH: Intermittent hypoxia, IA: Intermittent air.

A subjective histological assessment by an independent pathologist indicated no obvious differences between groups in PAS and trichrome staining, except for a variation in the amount of mesangial matrix in the glomeruli of the IH group that required further non-descriptive analysis. On the other hand, differences were evident in hematoxylin and eosin stained slides. Kidney sections from the IH group had a mild degree of glomerular congestion, mesangial expansion and pelvic inflammation. Fig 2 shows examples of kidney sections stained with hematoxylin and eosin (Fig 2A and 2B), PAS (Fig 2C and 2D) and Masson's trichrome (Fig 2E and 2F) stains.

### Glomerular area and MME

**Fig 3:**
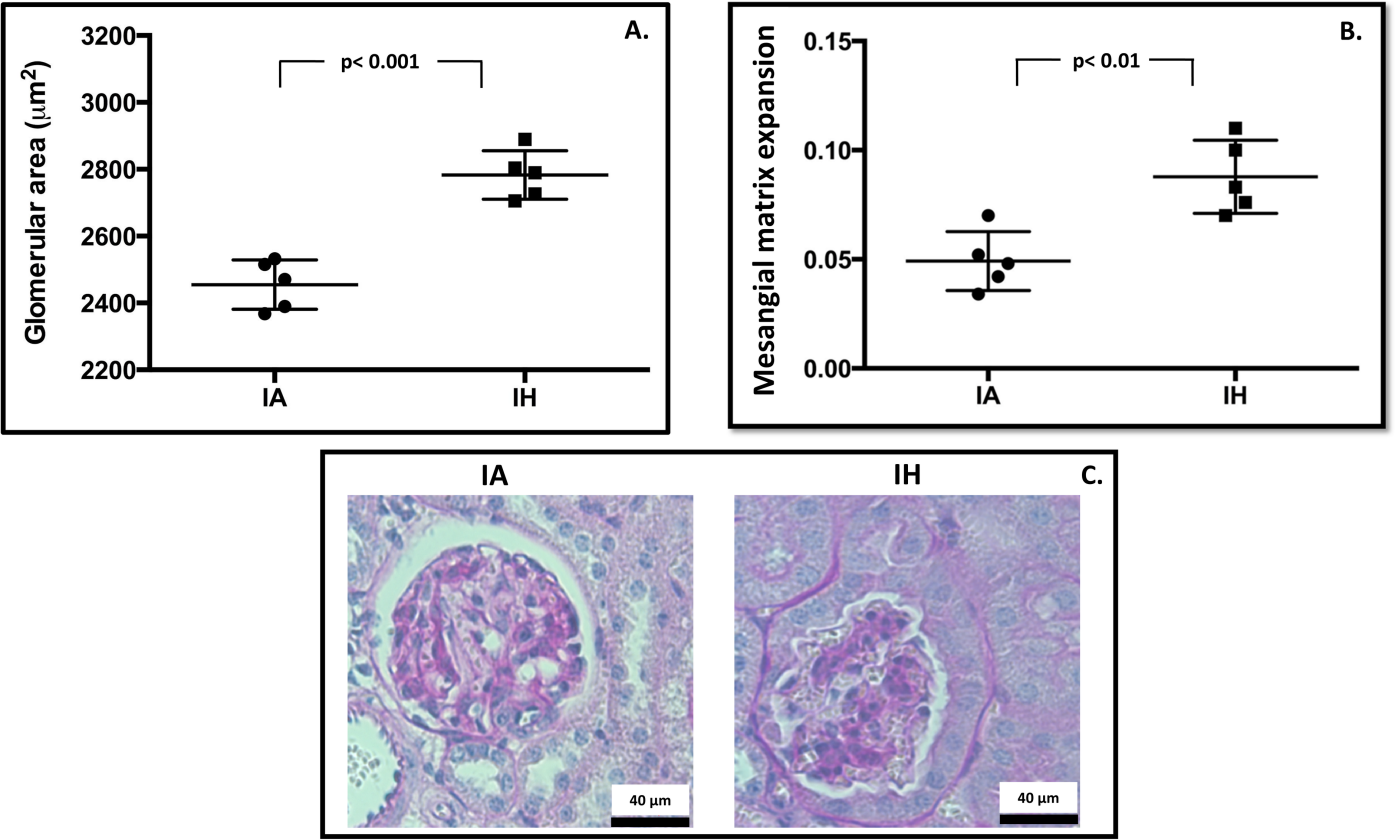
Histological evaluation. (3A) The glomerular tuft area was averaged from all available glomeruli per kidney section to give n = 1, using one kidney section per animal. (3B) Mesangial matrix fraction was calculated as the ratio of PAS positive area to total glomerular tuft area and averaged from 50 randomly selected glomeruli per kidney section to give n = 1. (3C) Examples of PAS-stained kidney sections; images taken at 400x magnification. Mesangium indicated as PAS-stained (purple) nuclei-free area within the glomerular tuft area. IA: Intermittent air, IH: Intermittent hypoxia; unpaired t-test, (n = 5).

A minimum of 145 different glomeruli per kidney section was included in the analysis of glomerular tuft area. The glomerular area in mice exposed to IH was increased by 13.4% (Fig 3A); the average glomerular tuft area in the IH group was 2782.84 ± 72.68 μm^2^ (95% CI: 2692–2872 μm^2^) compared to an average glomerular tuft area of 2454.98 ± 73.59 μm^2^ (95% CI: 2362–2546 μm^2^) (p< 0.001) in the IA control group. Further examination of PAS stained kidney sections indicated an expansion of mesangial matrix in the IH group by 1.8-fold compared to controls (Fig 3B). The mean fraction of mesangial matrix to glomerular tuft area in IH exposed mice was 0.087 ± 0.015 (95% CI: 0.032–0.065), compared to 0.049 ± 0.012 (95% CI: 0.064–0.108) in controls (p< 0.01).

### Immunohistochemistry

**Fig 4:**
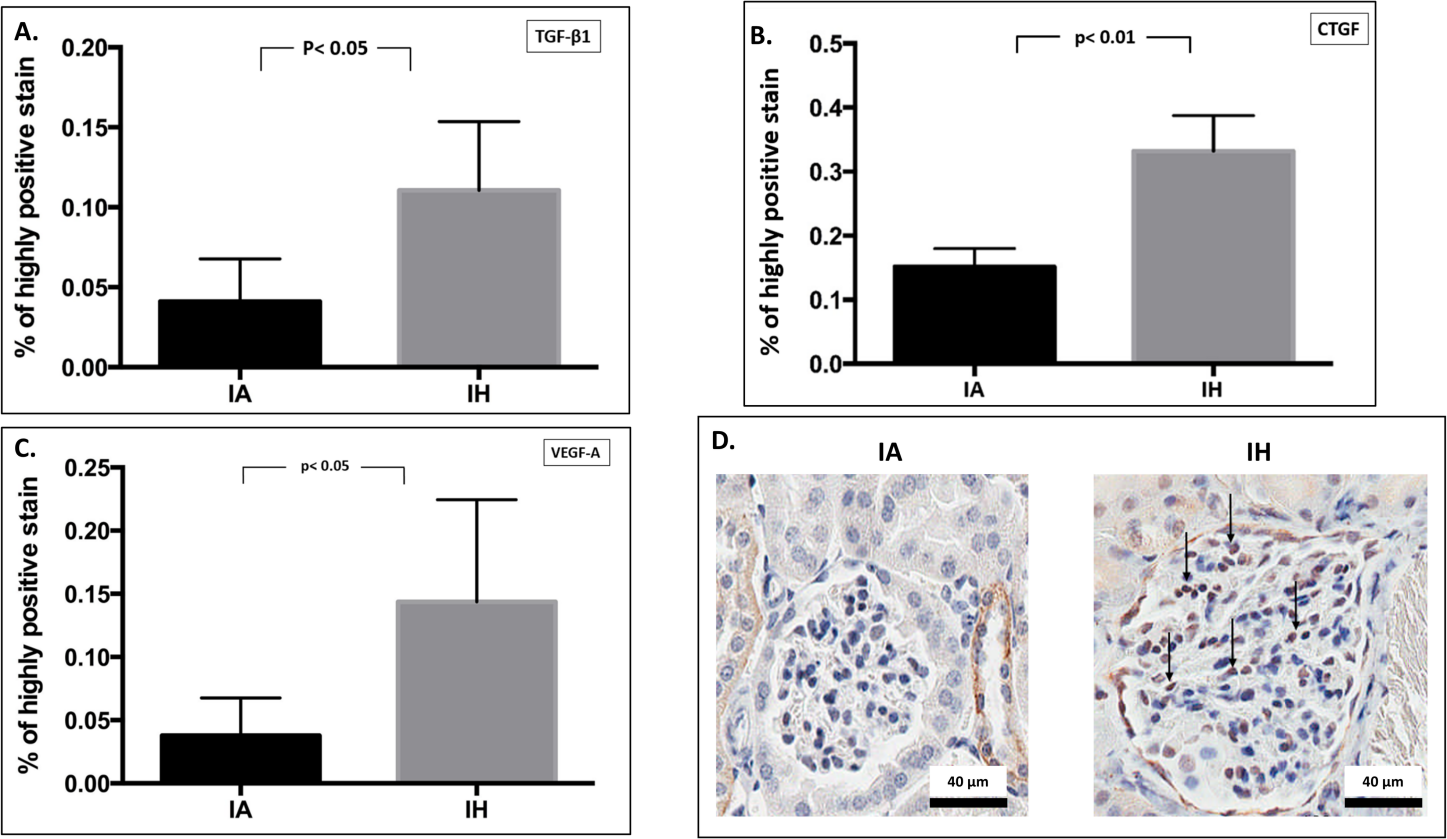
Glomerular expression of growth factors by immunohistochemistry. Semi-quantitative analysis of glomerular expression of TGF-β1 (4A), CTGF (4B) and VEGF-A (4C) proteins by immunohistochemistry. Fig 4D indicates an examples of glomerular protein (CTGF) expression by microscopic viewing. Images taken at 400x magnification; nucleus indicated as blue, while brown staining indicates antigen-antibody reaction inside glomerular cells. Arrows indicate glomerular protein localization. IA: Intermittent air, IH: Intermittent hypoxia; unpaired t-test, (n = 5).

We examined a number of profibrotic proteins to explore their role in MME secondary to IH. The glomerular expression of TGF-β1 and one of its essential downstream proteins, CTGF, were significantly increased by 2.7 (p< 0.05) and 2.2 (p< 0.01) fold respectively in response to IH (Fig 4A and 4B). Moreover, the glomerular expression of VEGF-A was significantly increased by 3.7-fold (p< 0.05) in the IH group compared to control IA group (Fig 4C). Table 2 summarizes the differences in glomerular protein expression between IH and IA groups.

**Table 2:** Changes in glomerular proteins expression by immunohistochemistry.

Protein	IA*	95% CI	IH*	95% CI	Change from control	p value
**TGF-β1**	0.041 ± 0.03	0.008–0.074	0.11 ± 0.04	0.057–0.164	2.7 X	< 0.05
**CTGF**	0.15 ± 0.03	0.116–0.186	0.33 ± 0.05	0.263–0.401	2.2 X	<0.01
**VEGF-A**	0.037 ± 0.03	0.0007–0.074	0.14 ± 0.08	0.043–0.243	3.8 X	<0.05

*: Expressed as mean % of highly-positive DAB chromogen ± SD, DAB: 3,3′-diaminobenzidine, IA: Intermittent air (Control), IH: Intermittent hypoxia, CI: confidence interval, TGF-β1: transforming growth factor-β1, CTGF: connective tissue growth factor, VEGF-A: vascular endothelial growth factor-A.

### Western blotting

**Fig 5:**
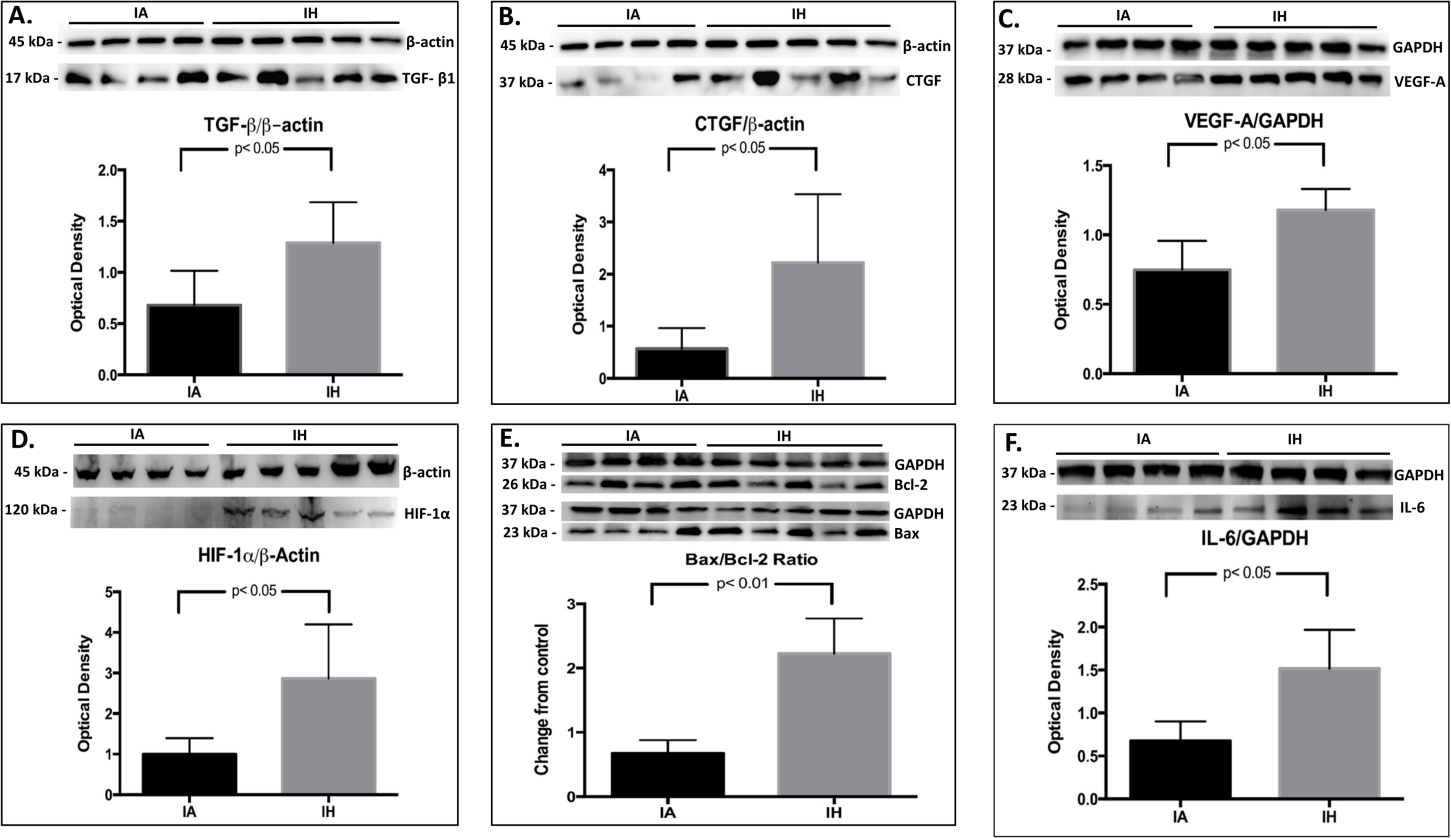
Proteins semi-quantification in kidney cortex by western blotting. Semi-quantitative measurements of TGF-β1 (5A), CTGF (5B), VEGF-A (5C), HIF-1α (5D) and Bax/Bcl-2 (5E) proteins in kidney cortex. Protein level was measured from kidney cortex tissue lysate. kDa: Kilodalton, IA: intermittent air, IH: intermittent hypoxia; unpaired t-test, (n = 4–5).

Cortical TGF-β1, CTGF and VEGF proteins were semi quantified by western blotting analysis. Compared to the IA group, the expression of TGF-β1, CTGF and VEGF-A proteins in IH-exposed mice was greater by 1.9, 4.0 and 2.6-fold respectively (p< 0.05 for all) (Fig 5A, 5B and 5C). The cortical expression of HIF-1α protein was also higher in the IH group by 2.9 times (p< 0.05) (Fig 5D). Finally, the ratio of apoptotic (Bax)/antiapoptotic (Bcl-2) proteins was 2.4 fold higher in IH-exposed mice (p< 0.01) (Fig 5E). Table 3 summarizes cortical protein expression in IH and IA groups.

**Table 3:** Changes in cortical proteins expression by western blotting.

Protein	IA*	95% CI	IH*	95% CI	Change from control	p value
**TGF-β1**	0.68 ± 0.33	0.35–1.01	1.27 ± 0.39	0.93–1.61	1.87 X	< 0.05
**CTGF**	0.57 ± 0.39	0.18–0.97	2.27 ± 1.33	1.10–3.43	3.98 X	< 0.05
**VEGF-A**	0.75 ± 0.21	0.54–0.95	1.18 ± 0.15	1.05–1.31	1.57 X	< 0.05
**HIF-1α**	1 ± 0.40	0.61–1.39	2.91 ± 1.42	1.67–4.16	2.91 X	< 0.05
**Bax**	0.62 ± 0.26	0.38–0.87	1.81 ± 0.76	1.15–2.48	2.92 X	< 0.05
**Bcl-2**	0.92 ± 0.24	0.69–1.15	0.81 ± 0.28	0.57–1.06	0.88 X	NS
**Bax/Bcl-2**	0.68 ± 0.20	0.48–0.88	2.23 ± 0.55	1.75–2.71	2.42 X	< 0.01

*: Expressed as the mean expression of protein band/expression of β-actin or GAPDH housekeeping protein ± SD, IA: Intermittent air (Control), IH: Intermittent hypoxia, CI: confidence interval, TGF-β1: transforming growth factor-β1, CTGF: connective tissue growth factor, VEGF-A: vascular endothelial growth factor-A, HIF-1α: hypoxia inducible factor-1α, Bax: Bcl-2-associated X, Bcl-2: B-cell lymphoma-2.

### Fluorometric *in situ* cell death detection

**Fig 6:**
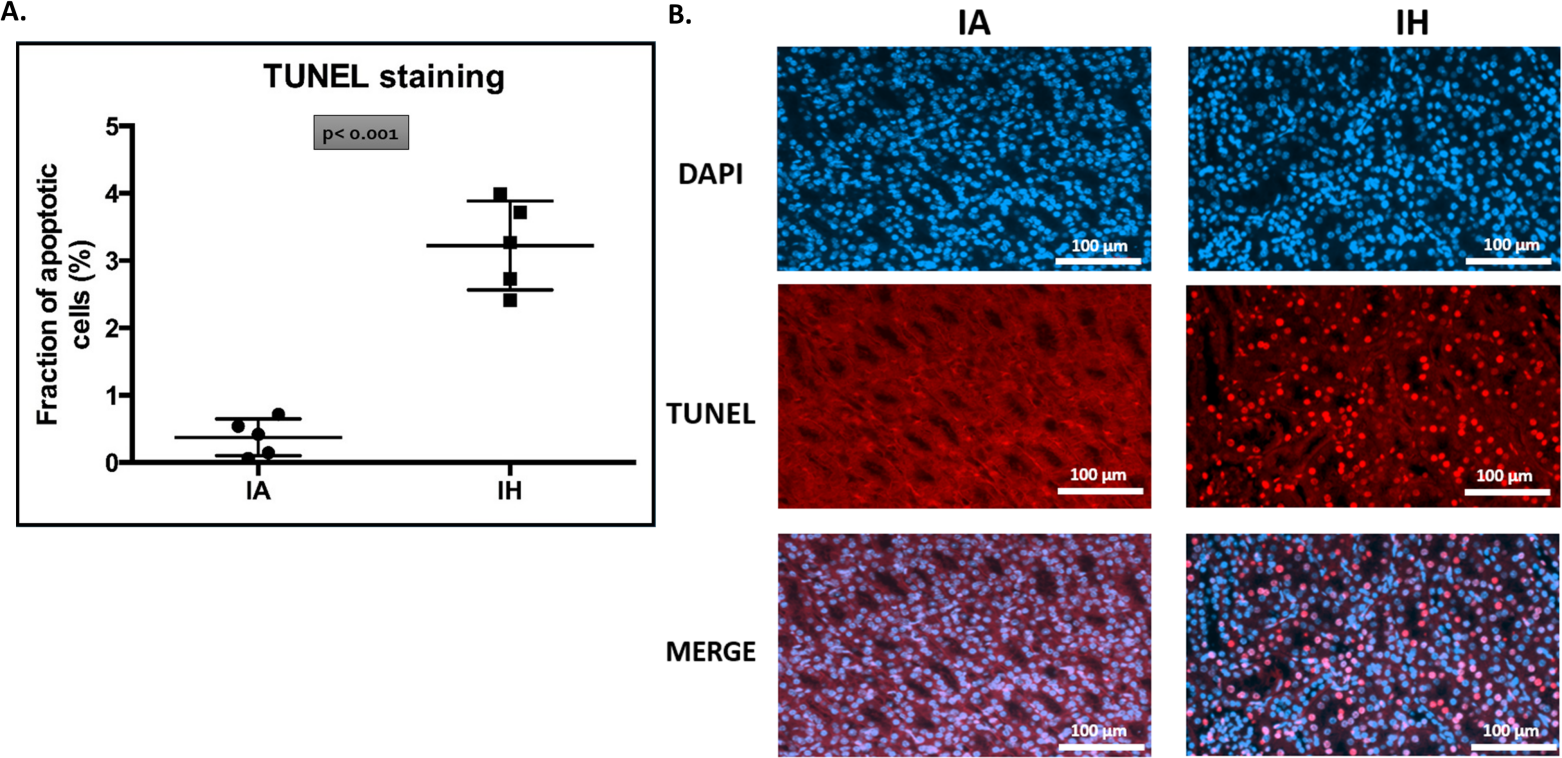
Fluorometric *in situ* cell death detection (TUNEL staining). Five paraffin fixed kidney sections from each group underwent *in situ* cell death detection (DNA-strand breaks) using a fluorometric TUNEL staining (red fluorescence) protocol. Slides were counter-stained with DAPI (blue fluorescence) for nuclear detection. The fraction of apoptotic cells was calculated as the number of TUNEL-positive nuclei to the total nuclei per image. Four different images were taken at (200x magnification) from each kidney section to give n = 1. Fig (6A) shows the mean fraction of TUNEL staining-positive cells to the number of available cells. Fig (6B) shows examples of the distribution and amounts of TUNEL positive cells by microscopic viewing. IA: intermittent air, IH: intermittent hypoxia; unpaired t-test, (n = 5).

Five paraffin-embedded kidney sections from each group underwent TUNEL-staining for detection of cells with DNA damage, and counterstained with DAPI for detection of nuclei. The percentage of TUNEL-positive nuclei in the IH group was 3.2 ± 0.67% (95% CI: 2.7–3.8%) compared to 0.38 ± 0.27% (95% CI: 0.14–0.61%) in the IA control group (p< 0.001) (Fig 6A).

### Renal function index

**Fig 7:**
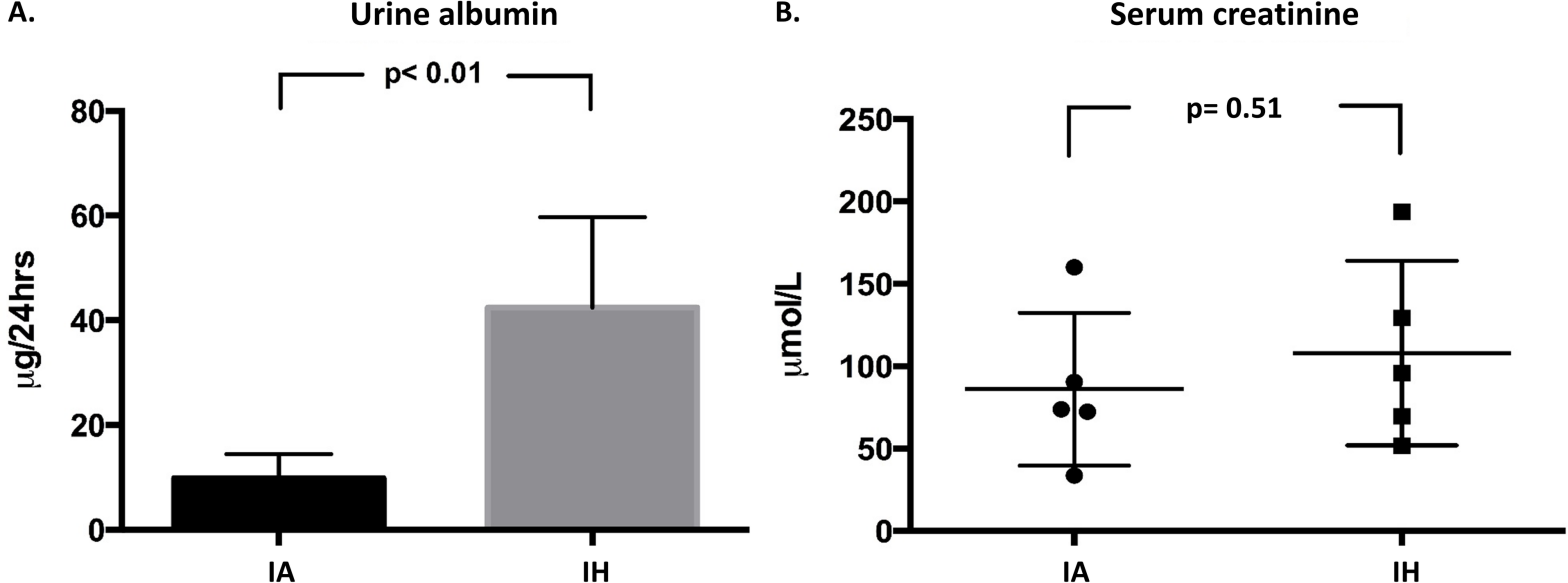
Renal function index. Urinary albumin excretion in 24 hours (7A); albumin concentration in urine sample was measured by a standard ELISA method and then normalized to the volume of urine excreted. Serum creatinine was measured by an enzymatic assay and expressed as a concentration in an 8 μl plasma sample (7B). IA: Intermittent air, IH: Intermittent hypoxia; unpaired t-test, (n = 5).

An average of 0.9 ± 0.13 ml and 0.86 ± 0.22 ml of urine was collected from IH-exposed and control mice respectively (p = 0.45). The average 24-hour urinary albumin excretion in the IH-exposed mice was 43.4 ± 16 μg (95% CI: 29.3–57.5 μg), while in control mice urinary albumin excretion averaged 9.7 ± 4.6 μg (95% CI: 5.7–13.8 mg) in 24-hours (p< 0.01) (Fig 7A). In contrast, the average serum creatinine level in IH-exposed mice was 108.02 ± 56.1μmol/L, compared to 86.1 ± 46.4 μmol/L in the IA control mice (p = 0.51) (Fig 7B).

## Discussion

Our study shows for the first time that IH causes glomerular hypertrophy and expansion of the glomerular mesangial matrix in a mouse model of OSA. We also show that mice exposed to IH have increased expression of glomerular TGF-β1, CTGF and VEGF-A proteins. In addition, there was a significant increase in renal cellular apoptosis after 2-months of IH. Our data also shows that IH-exposed mice excrete higher amounts of urinary albumin compared to control mice, suggesting that there was mild disruption of glomerular filtration in these mice, possibly related to glomerular hypertrophy [24]. There was no evidence of severe renal functional damage secondary to IH, at least based on serum creatinine data (Fig 7B).

Glomerular hypertrophy is associated with glomerulosclerosis, where the interplay of hemodynamic and various growth factors determines the balance between glomerular matrix accumulation and degradation [25]. Although MME occurs in diabetes [26], other diseases such as hypertension also cause glomerular hypertrophy in humans [27]. Nevertheless, factors such as obesity [28] and hyperuricemia [29] are also associated with glomerular hypertrophy. Increases in glomerular profibrotic cytokines is suggested to be the principal pathway in the pathogenesis of MME or glomerular hypertrophy. With respect to renal pathophysiology, TGF-β1 is a pleiotropic cytokine secreted by glomerular mesangial cells and podocytes in response to common fibrogenic stimuli [29], and is considered the primary fibrogenic growth factor in the pathogenesis of renal fibrosis [30–33]. In addition, TGF-β1 is also a major inducer of CTGF in renal fibrosis [34], where excessive production of glomerular CTGF is implicated in glomerulosclerosis and thickening of glomerular basement membrane [35–36].

Several epidemiological studies suggest a possible relationship between sleep apnea and declining kidney function [37–39]. A retrospective cohort study reported that about 30% of patients with sleep-related breathing disorders were also diagnosed with chronic kidney disease (CKD), which was significantly greater than the prevalence of CKD in the healthy population [37]. However, the apnea-hypopnea index was not a significant determinant of CKD in sleep-related breathing disorder patients [38]. Another retrospective cross-sectional study detected significant reductions in glomerular filtration rates as the severity of OSA increased [39]. The prevalence of diabetes and hypertension is also significantly higher in patients with severe OSA [39]. On the other hand, sleep apnea is a significant comorbidity in patients with CKD as shown in a prospective study where the prevalence of sleep apnea (predominantly obstructive) increased significantly with declining kidney function. This study reported that 57% of patients with end-stage renal disease had sleep apnea, compared to 41% of patients with CKD not on dialysis and 27% of patients with glomerular filtration rates of ≥60 ml/min [40].

Suggested mechanisms linking OSA and CKD include, but is not limited to, activation of the renin-angiotensin-aldosterone system (RAAS), increased sympathetic regulation and elevations in systemic and local reactive oxygen species [41–42]. The effect of nocturnal hypoxia is common in OSA patients and cannot be overlooked in the context of OSA-related CKD. A recent cohort study reported that OSA patients with nocturnal hypoxia (defined by oxygen saturation< 90% for> 12% during night-time monitoring) were at significant risk for accelerated loss of kidney function, with an adjusted odds ratio of 2.89 (95% CI: 1.25–6.67) [43]. In addition, another study reports that RAAS can be influenced by nocturnal hypoxemia in OSA patients, where patients with severe hypoxemia (mean SaO_2_≤ 90% during overnight) have significantly greater renal RAAS activity compared to moderate hypoxemia patients (SaO_2_≥ 90%) and control subjects [44]. Interestingly, continuous positive airway pressure (CPAP) therapy reverses the increased activity of RAS in patients with OSA, as shown by a recent clinical trial where CPAP therapy increased renal plasma flow and significantly reduced plasma aldosterone and urinary protein excretion in non-diabetic normotensive OSA patients [45].

Although our study focusses on glomerular signs of injury secondary to IH, we have not examined specific markers of tubular structural or functional injury. The chronic hypoxia hypothesis states that hypoxia is a key player in inducing primary glomerular injury and that it creates a hypoxic tissue environment that eventually triggers tubular injury [46]. In fact, renal tissue hypoxia is a major pathological mechanism in triggering several renal pathologies such as hypertensive and diabetic nephropathies [47]. Renal tissue hypoxia induced in rats by administering dinitrophenol (a mitochondrial uncoupler that increases oxygen consumption) increases markers of renal injury similar to those occurring in hypertensive and diabetic nephropathy, e.g. increased renal tissue oxygen consumption and urinary protein excretion [48].

Despite growing clinical evidence of OSA-related CKD, the direct effect of OSA on kidney structure and function is largely unexplored, leaving the pathological association between OSA and CKD unclear. A possible explanation for this is that the majority of OSA patients frequently present with multiple comorbidities, making it difficult to establish the direct effects of OSA on CKD. Studies on animal models may therefore be a useful approach to examine the direct effects of OSA on kidney pathology. For example, a study in mice using similar study design as in our study reported that 8 weeks of IH upregulated a number of inflammatory and profibrotic proteins (including CTGF) in kidney tissues, suggesting a direct destructive effect of IH on kidneys [49]. However, unlike the urinary protein analysis data reported in our study, Sun et al reported no differences in 24-hour excretion of urinary proteins [49]. Others report that metallothionein (potent antioxidant) knock-out mice were more prone to renal damage when exposed to 8 weeks of IH [50]. Another recent study reported that 2 weeks of IH significantly increased plasma VEGF in mice [51]; interestingly, mouse macrophages exposed to IH *in vitro* also have significant increases in VEGF expression [51]. Such studies not only strengthen a clear pathological linkage between OSA and the renal system, but also illustrate potential treatment strategies to avoid OSA-related renal consequences.

The animal model of IH we use has also been used to investigate other sleep apnea-related comorbidities such as cancer. It is important to note variations in the severity and duration of IH in this model; for instance, Almendros and colleagues reported a significant increase in lung epithelial TC1 cell tumors in mice exposed to levels of IH that are close in its severity to the hypoxic levels in our study (FIO_2_ = 6%), but with a duration of only 28 days [11]. Another study by the same group reported increased melanoma lung metastasis in mice after being exposed to IH for 30 days using FIO_2_ of 5%, but for only 6 hours per day [52].

Our study has a number of strengths; the use of an animal model excludes the effects confounding variables such as pre-existing hypertension, diabetes, and obesity that are potential confounders in human studies. However, our study also has a number of limitations. First, the number of mice studied was relatively small. Second, our mouse model is not a perfect representation of human OSA. For example, patients with OSA tend not to have such severe desaturation, and suffer from hypercapnia during events as opposed to hypocapnia [53]. However, IH is one of the key components of OSA, and this model has been used extensively in the literature especially in terms of elucidating cardiometabolic complications associated with OSA. Another important limitation of this model is that it does not allow for easy recording of factors usually accompany sleep apnea, such as sleep fragmentation. The recurrent arousals combined with hypoxemia/reoxygenation can activate the sympathetic nervous system, oxidative stress, and inflammation; these may represent important components in the increased risk of cardiovascular diseases including myocardial infarction and stroke in OSA patients [54]. Although mice exposed to IH in our study did not show any signs of diabetes and obesity, we were unable to reliably monitor blood pressure during the wake and sleep cycles of mice. Others have reported increases in the mean arterial pressure (by ~14 mmHg) in a rat model of IH [55]. Therefore, we are unable to rule out the effects of secondary hypertension due to IH on glomerular alterations.

In summary, we show that kidneys are an important target for the harmful effects of IH. We provide evidence for glomerular hypertrophy and MME in response to IH, with simultaneous increases in glomerular TGF-β1, CTGF and VEGF-A proteins. Taken together, these findings allow for a better understanding of the mechanisms by which IH induces renal damage, and suggest potential targets for mitigating the potential kidney damage related to OSA.

## Supporting information

S1 Table Table of 3 point scale used for the general monitoring of animals. (XLSX)
